# Home Sweet Home: The Impact of Lifestyle on a Cat’s Approach to Impossible Tasks in the Home Environment

**DOI:** 10.3390/ani13162679

**Published:** 2023-08-20

**Authors:** Anna Scandurra, Alfredo Di Lucrezia, Biagio D’Aniello, Claudia Pinelli

**Affiliations:** 1 Department of Biology, University of Naples Federico II, 80126 Naples, Italy; alfredo.dilucrezia@unina.it (A.D.L.); biagio.daniello@unina.it (B.D.); 2 Department of Environmental, Biological and Pharmaceutical Sciences and Technologies, University of Campania Luigi Vanvitelli, 81100 Caserta, Italy; claudia.pinelli@unicampania.it

**Keywords:** cat, home environment, impossible task, indoor-outdoor, living condition, well-being

## Abstract

**Simple Summary:**

The effect of living style (i.e., indoor/outdoor) on domestic cats was studied. The behavior of the domestic cats with different living styles (e.g., indoor only and indoor/outdoor) was observed during the impossible task paradigm, a test in which the cats learn to open an apparatus to obtain a food reward and, immediately after, experienced the expectancy of violation in a trial in which the apparatus was blocked. Tests were carried out in the house where the cats lived and the only person present with the cat during the test was the owner. The results show the effect of living style and age on the problem-solving approach of domestic cats. Indoor/outdoor cats spent less time interacting with the apparatus and showed stress behaviors sooner compared to indoor cats. Research on this topic can be useful for improving the welfare of domestic cats.

**Abstract:**

Cat welfare is a topic of growing interest in the scientific literature. Although previous studies have focused on the effects of living style (i.e., indoor/outdoor) on cat welfare, there has been a noticeable dearth of analysis regarding the impact of lifestyle on cats’ inclination and mode of communication with humans. Our research aimed to analyze the possible effect of lifestyle (e.g., living indoors only or indoor/outdoor) on cat–human communication. The cats were tested using the impossible task paradigm test, which consists of some solvable trials in which the subject learns to obtain a reward from an apparatus, followed by an impossible trial through blocking the apparatus. This procedure triggers a violation of expectations and is considered a useful tool for assessing both the decision-making process and the tendency to engage in social behaviors towards humans. A specific ethogram was followed to record the behavioral responses of the cats during the unsolvable trial. Our results show the effects of lifestyle and age on domestic cats, providing valuable insights into the factors that influence their social behaviors. Cats that can roam freely outdoors spent less time interacting with the apparatus compared to indoor-only cats. Additionally, roaming cats showed stress behaviors sooner following the expectancy of violation compared to indoor cats. The lifestyle of cats can influence their problem-solving approach while not affecting their willingness to interact with humans or their overall welfare. Future studies on this topic can be useful for improving the welfare of domestic cats.

## 1. Introduction

Cat domestication occurred about 10,000 years ago [1], when the wildcat (*Felis silvestris lybica*) probably began to prey on rodents in the proximity of human settlements [2]. The wildcat is known for its solitary hunter behavior and limited intraspecific sociability [3]. While domestic cats (*Felis silvestris catus*) retained numerous characteristics from their ancestors [4], they have undergone rapid evolutionary changes that enable them to adapt to and thrive within an intraspecific social system [3]. Domestic cats can occasionally form groups with other cats, depending on their living conditions and the presence of people that provide food for them. They can communicate with conspecifics belonging to their social group [5] but maintain some instinct in line with their wild ancestors, including the need to hunt, increased physical activity at night, and a territorial home range adequate for a solitary life [6]. Even though cats have undergone the domestication processes less intensely than dogs [1,7,8,9], they effectively share their environment with humans as dogs [6,9,10]. Although these felids do not cooperate with conspecifics to obtain food, they can use different types of signals [10], such as gaze, head movements, and body language, to obtain rewards from humans [9,11,12].

In recent years, scientific interest in the cognitive and social abilities of cats has been increasing. Studies demonstrated that cats may form an attachment bond with their human caregivers [13,14], although Potter and Mills [15] did not find evidence of secure attachment as has been described in dogs [16,17,18]. Domestic cats may be able to understand human emotional expressions [19,20], can recognize owners from their vocal cues [21], and they often rely on their owners for information about non-familiar stimuli, adapting their behaviors based on the owners’ emotional reactions in the “social referencing” process [22]. As shown in dogs [23,24], domestic cats are capable of perceiving information provided by humans through multimodal communication channels (i.e., acoustic and visual signals) and show faster responses when human signals are visual rather than acoustic [25]. It has recently been shown that cats are able to perceive human emotional chemosignals (e.g., the smell of human fear) and to implement behavioral responses consistent with the perceived smell (i.e., they increase stress levels in tested cats) [26].

Living with a cat can also benefit people. Several researchers have shown that sharing an environment and interacting with cats can reduce depression, anxiety, and introversion in people [27,28,29].

With the burgeoning interest in the cognitive and social capacities of cats within the scientific community [30,31,32,33,34], the primary aim of these investigations centers on feline welfare. It is still unclear what factors may be involved in the behavioral patterns of domestic cats and how these may affect their welfare in a human environment. Recent studies focused on the effects of living style (i.e., indoor/outdoor) on cat welfare. Living style may change depending on the country and the local culture [34], with some owners allowing their cats to live indoor-only or with free access to go outside. The indoor-only lifestyle was linked to more problematic behaviors (e.g., spraying, scratching the furniture, aggression towards people) than the indoor-outdoor option [27,35,36,37] and, generally, access to the outdoor environment may have positive outcomes for their well-being [33]. Despite these advantages, many owners limit outdoor access to protect cats from traffic accidents, ingesting poisoned food, wild animals, for fear of losing them or because, in some countries, outdoor cats are killed by law to protect wildlife [34]. On the other hand, recent research has provided evidence that cats kept exclusively indoors recorded higher scores in quality of life compared to cats with outdoor access, as reported by their owners [38]. Research in this field provided important information on the factors that affect the well-being of cats but has some limitations due to the different methodologic approaches (e.g., questionnaires or ethological tests) and the testing environments (e.g., shelters or laboratories, cat homes) in which they were performed [30,33,39].

Despite the abundance of research papers exploring various aspects of feline behavior, there has been a noticeable dearth of analysis regarding the impact of lifestyle on cats’ inclination and mode of communication with humans [31]. The first focus of the present research is to study the behavior of cats faced with an unsolvable problem in the presence of their owner in a home environment. In particular, cats were subjected to the impossible task paradigm, an experimental procedure that was successfully applied to several domesticated species beyond cats [9,40], such as dogs [41], horses [42], cattle [43], calves [44], and goats [45]. Specifically, Miklósi et al. [9] conducted a study comparing the social responses of cats and dogs toward humans. Their findings indicated that dogs exhibited a significantly stronger inclination towards engaging in social behavior compared to cats, whereas cats demonstrated a tendency to approach the task with a more autonomous approach. A study by Zhang et al. [37] demonstrated that, during the unsolvable test, cats exhibited a higher frequency of gaze alternation but displayed less interaction with their owner. Additionally, the cats spent longer durations gazing at their owner when the owner was attentive, as opposed to when the owner was inattentive. While both studies provide valuable insights into the social behavior of cats in engaging with humans during the impossible task paradigm, they have not considered the potential influence of ontogenetic factors that could introduce biases in their propensity for social interaction with humans. Knowing which factors can influence the behavior of domestic cats in the home environment and how they affect their behavior can improve human–cat relationships and provide necessary information about the well-being of cats [31,33]. In our study, we focused on the lifestyle of domestic cats, evaluating the behavioral responses of cats that did not have access to the outside of the house and comparing their behavior with those of cats that lived in the house but could also have free access to go outside. Based on the possible exposure of the roaming cats to different challenges that they have to solve autonomously, we hypothesize that, when presented with an expectancy violation in an impossible task, cats allowed to roam will exhibit increased persistence in attempting to solve the task and a reduced inclination for social communication with their owners.

## 2. Materials and Methods

### 2.1. Subjects

A total of 60 domestic cats (40 males and 20 females) were recruited and involved in the test. The age of animals ranged from 2 to 16 years old (mean age ± SD: 5.70 ± 3.40 years). The cats in our study were classified into two groups based on their lifestyle: those that lived exclusively indoors (henceforth indoor cats) and those that lived indoors but were allowed to roam outdoors (henceforth roaming cats). We selected 42 indoor cats (17 females and 25 males, mean age ± SD: 5.73 ± 3.70 years) and 18 roaming cats (3 females and 15 males, mean age ± SD: 5.61 ± 2.70 years). Volunteers were recruited through personal contacts and advertisements on the internet, in parks, and at veterinary surgeries.

### 2.2. Apparatus and Location

The experimental apparatus consisted of a plastic food container placed on a rectangular wooden platform (38 × 15 cm). During the solvable trials, the lid of the container was fixed upside down on the platform with screws, and the container was simply placed upside down on the lid. In contrast, during the unsolvable trial, the container was locked onto the lid. The wooden platform was fixed to the floor with double-sided adhesive tape. It has been shown that cats are easily stressed by changes in their home environment, even if these involve changes or shifts in furniture [46]. To ensure that the cats were tested in a familiar and comfortable environment, the experiments were conducted within the cats’ own homes. A specific room, which the cats typically had access to, was chosen for the testing procedure. During the test, the room was closed off to prevent interference from outside sources and to create a controlled environment. Additionally, the room was devoid of distracting elements, with only the necessary materials for the testing procedure present.

### 2.3. Experimental Procedure

**Figure 1:**
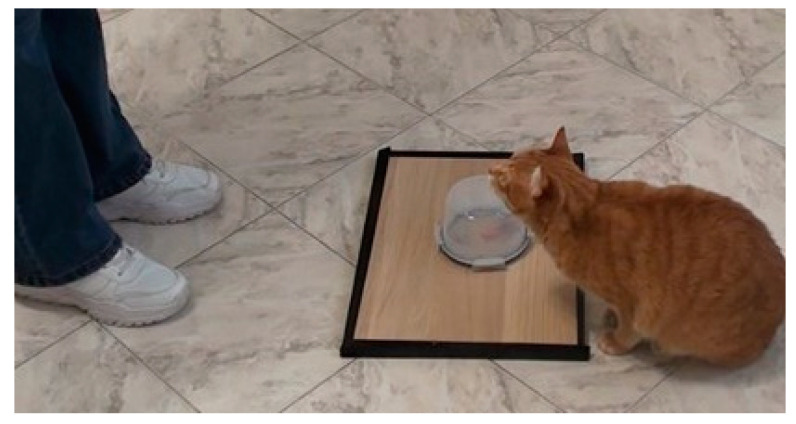
Experimental area with the owner, apparatus, and cat during the unsolvable trial.

During the entirety of the procedure, the owner served as the sole individual present. The experimenter provided the owner with detailed instructions on how to experiment, including the specific procedures to follow. However, the overall purpose of the study was not disclosed to the owner, ensuring that their actions and observations were unbiased and not influenced by prior knowledge of the study’s objectives. Owners were instructed not to feed their cats in the 4 h before the test. Since food palatability plays an important role in cats [46], each owner was asked to use their cat’s favorite food. The cats’ food motivation was ascertained by offering a few pieces of food to the cat before the test began. The impossible task paradigm consisted of two different phases: the first one was named “solvable phase” and comprised three consecutive solvable trials; the second phase, named “unsolvable phase”, involved only one trial to which the cat was subjected immediately after the end of the previous phase. During the solvable trials, the owner placed the food on the lid and covered it with the food container, inviting the cat to recover it. The trial could be solved by moving away the container with the muzzle or paw. The cats that failed to solve the three trials were not subjected to the unsolvable phase and were excluded from the final sample. During the unsolvable trial, the owner locked the food container on the lid and assumed a stationary position at about 30 cm from the apparatus (Figure 1), staring straight ahead and ignoring the cat for the duration of the test trial (60 s). All applicable international, national, and/or institutional guidelines for the care and use of animals were followed. All experimental procedures comply with the ethical standards under EU Directive 2010/63/EU for animal testing.

### 2.4. Data Analysis

A Sony^®^ HDR-PJ260VE (Sony, Tokyo, Japan) camera was utilized to record the experimental procedure. The videos were analyzed by a trained researcher, and the behaviors during the unsolvable phase were coded using Solomon Coder^®^ beta 19.08.02 (ELTE TTK, Budapest, Hungary). Behaviors were grouped in different categories according to a specific ethogram (Table 1). A second independent researcher coded 25% of the video (i.e., 12 video) for inter-observer reliability. The videos were encoded in blinded mode: both coders did not know neither the sex nor the group of the cats before the end of the data collection. Pearson correlation was used to compare the data obtained for each behavioral categories by the two coders, both for the duration and the latency. For the duration, the correlation was high: apparatus, r = 0.993, *p* < 0.001; owner, r = 0.982, *p* < 0.001; others, r = 0.908, *p* < 0.01; stress, r = 0.915, *p* < 0.005. For the latency, the correlation was the sequent: apparatus, r = 0.899, *p* < 0.005; owner, r = 0.997, *p* < 0.001; others, r = 0.878, *p* < 0.01; stress, r = 0.905, *p* < 0.01. Considering the results obtained for the inter-observer reliability, the data of the first coder were used for all statistical analyses.

The parameters of duration (expressed in seconds) and latency (i.e., seconds elapsed from the beginning of the trial to the first occurrence) were collected for all behaviors. The Kolmogorov–Smirnov test indicated the non-normal distribution for most of the data; thus, a nonparametric approach was adopted. To explore differences in behaviors based on lifestyle, the Mann–Whitney U test was used to compare indoor and roaming cats. Furthermore, to control for the potential effect of sex and age, the data were analyzed with a Generalized Linear Model (GzLM). Each behavior was incorporated into the models as the dependent variable in the analysis, utilizing a Tweedie distribution. This choice of distribution was made to account for the modeling of our data, which were often skewed. On the other hand, Tweedie distribution accommodates a wide range of distributional shapes. The fixed factors included in the models were living style (i.e., indoor or roaming) and sexes (i.e., female or male), while age was utilized as a covariate within the model. The *p*-value for the omnibus test and α values were set at 0.05. All analyses were performed with SPSS^®^ Statistic Software (IBM^®^ Corporation, Armonk, NY, USA, version 26.0.0.1).

## 3. Results

**Figure 2:**
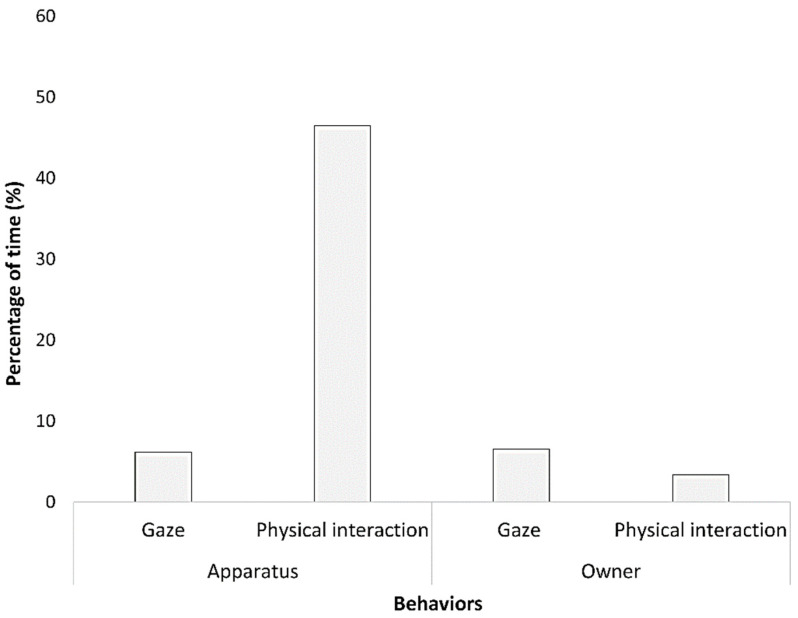
Graphical representation of the behaviors (gaze and physical interaction) directed to apparatus and owner recorded for all cats in the unsolvable trial. Behaviors were expressed as percentage of the time.

All cats involved in the test showed interest in the food during the solvable tasks and passed to the unsolvable phase. Globally, the most expressed behavior during the unsolvable trial was the physical interaction with the apparatus (46.48% of the time; Figure 2). Cats gazed at the apparatus for 6.17% of the time, while they gazed at their owner for 6.57% of the time during the test. Physical interaction with the owner was registered for 3.36% of the time. In the remaining time (37.42%), cats performed behaviors not directly related to task resolution. Stress behaviors were recorded for 5.75% of the time. In particular, the stress behavior most expressed by all cats was self-grooming.

**Figure 3:**
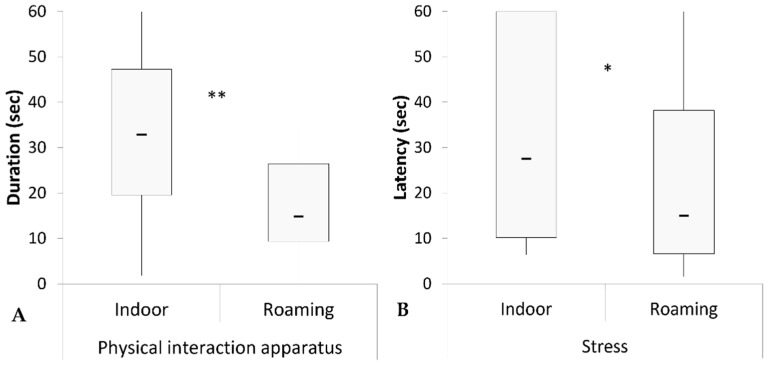
Graphical representation of physical interaction with the apparatus (duration, (**A**)) and stress behaviors (latency, (**B**)) recorded for indoor and roaming cats in the unsolvable trial. Black rectangles represent medians; boxes indicate the quartiles from 25 to 75%; thin vertical lines show minimum and maximum values. * *p* < 0.05, ** *p* < 0.01.

The comparison between two different lifestyle groups revealed that indoor cats physically interacted with the apparatus for longer than roaming cats (indoor: median = 32.8 [min = 1.8, max = 60]; roaming: median = 14.8 [0, 34.4]; Mann–Whitney U = 167, *p* < 0.001; Figure 3A). Indoor cats showed stress behaviors later than roaming cats (indoor: median = 27.5 [6.4, 60]; roaming: median = 14.9 [1.6, 60]; Mann–Whitney U = 233.5, *p* = 0.019; Figure 3B).

The GzLM analyses revealed the significant main effect of living style on the physical interaction with the apparatus. Indoor cats exhibited a higher probability of engaging physically with the apparatus for a longer period compared to roaming cats (β = 0.663; χ^2^ = 14.233; *p* < 0.001). They also showed a faster response, interacting physically with the apparatus with a lower latency compared to roaming cats (β = −2048; χ^2^ = 18.781; *p* < 0.001). Furthermore, indoor cats had a lower probability of displaying stress behaviors during the unsolvable trial compared to roaming cats (β = −1097; χ^2^ = 8.414; *p* = 0.004). The GzLM models revealed that age had a significant effect on the behavior of gazing at the apparatus. Specifically, older cats have a higher probability of gazing at the apparatus for a longer duration compared to younger cats (β = 0.111; χ^2^ = 8.165; *p* = 0.004). Age also had a significant effect on the duration and latency of physical interaction with the apparatus. Older cats demonstrated a higher probability of physically interacting with the apparatus for a shorter duration (β = −0.062; χ^2^ = 7.53; *p* = 0.006) compared to younger cats. Moreover, older cats had a higher probability of physically interacting with the apparatus later from the beginning of the unsolvable trial (β = 0.175; χ^2^ = 7.24; *p* = 0.007) compared to younger cats. No significant sex differences were observed in our sample, and there were no other significant effects on the remaining behavioral parameters.

## 4. Discussion

Cats are one of the most common pets living in the anthropogenic niche [10], where they can experience a wide range of living conditions and management [47]. Our research aimed to study the possible effect of lifestyle on cats’ tendency to engage in social behaviors with humans using the impossible task paradigm. The experimental procedure consists of some solvable trials in which the subject learns to obtain a reward from an apparatus in a simple way, followed by an impossible phase through blocking the apparatus. This procedure triggers a violation of expectations and is a useful tool for assessing both the tendency to engage in social behaviors towards humans and the decision-making process. In this study, we focused on the potential impact of lifestyle on cats’ tendency to engage in social behaviors with humans in the impossible task paradigm. Specifically, we conducted a comparison between cats that were allowed to roam freely outdoors and a group of cats that did not have free access to the outside environment. Cats that are allowed to roam freely outdoors obviously encounter a higher frequency of problem-solving situations compared to cats that do not have the same roaming habitude. This exposure to various challenges can contribute to their ability to solve problems more effectively. Additionally, roaming cats may have fewer opportunities for social communication with humans due to their increased independence and interactions with the outdoor environment. Our findings indicate that lifestyle had an impact on the duration of physical interaction with the apparatus and the latency of stress behaviors. Specifically, we observed that roaming cats spent less time interacting with the apparatus compared to indoor cats. Additionally, roaming cats exhibited stress behaviors at an earlier latency compared to indoor cats. These results present a challenge to our working hypothesis, especially considering that there were no observed differences in behaviors directed toward the owners between the two groups. In this context, the co-expression of these behaviors in the roaming group suggests a more rapid realization of the task’s impossibility. This could explain the shorter latency in the manifestation of stress signals. The outdoor exploration experiences and environmental enrichment in the roaming group may contribute to their decreased interest in the task once they become aware that it cannot be solved. On the other hand, indoor cats, who may experience boredom and a lack of stimulation [30], may have exhibited greater physical engagement in attempting to solve the task. This behavior could be driven by the lower level of stimuli and environmental enrichment present in their home environment. These findings align with a previous study demonstrating that indoor cats exhibited a stronger interest in various stimuli compared to roaming cats [48]. Indoor cats may perceive the task as a novel experience that stimulates their curiosity, thereby increasing the timing of interaction. This is because indoor cats typically do not engage in predatory behavior, except during play [48], and rely on humans for food provision [49]. The contrasting approaches to the task involving food unavailability could be influenced by the disparity in predatory behavior and independent food provision between indoor and roaming cats.

In our study, we found that older cats had a higher probability of engaging in shorter physical interactions with the apparatus compared to younger cats. This finding contrasts with a previous research study which showed that older cats approached the container more frequently than younger cats in the unsolvable task paradigm aimed at studying social referencing in cats. However, our results align with another research demonstrating that younger cats exhibit more active and explorative behaviors [50]. These discrepancies may be due to variations in the specific tasks used and the behavioral aspects being examined.

## 5. Conclusions

Our findings shed light on the intricate interplay between lifestyle, age, and behavioral tendencies in domestic cats, providing valuable insights into the factors that influence their social behaviors. It appears that roaming behavior in cats can influence their problem-solving approach while not affecting their willingness to interact with humans or their overall welfare, despite the potential hazards associated with roaming. Research aimed at improving the welfare of cats living with humans holds great interest, not only for feline well-being but also for enhancing human quality of life [27,28,29]. Future research should delve deeper into the specific factors that impact cats’ well-being in the home environment, considering that well-being may depend on individual factors or combinations thereof [47].

## Figures and Tables

**Table 1:** Ethogram was applied for the study. All behaviors were mutually exclusive, except for stress behaviors that can be recorded at the same time as other behaviors.

Category	Behavior	Description
Apparatus	Gaze apparatus	Gazing at the apparatus from a stationary point of view
	Physical interaction apparatus	Physical contact with the apparatus in trying to solve the task
Owner	Gaze owner	Gazing at the owner from a stationary point of view
	Physical interaction owner	Physical contact with the owner
Others	All behaviors are not aimed at solving the task.	Visual or olfactory exploration, out-of-sight (the cat is not visible in the video), and vocalizations
Stress	Yawning	Opening the mouth widely
	Scratching	Scratch themselves with the hindleg
	Shaking head	Moving head side to side in a rapid manner
	Shaking leg	Moving limb sideways and back rapidly
	Self-grooming	Licking fur, wiping head region with paw after licking
	Oral behavior	Licking nose and oral region

## Data Availability

The data presented in this study is available on request from the corresponding author.
